# TGFβ Activated Kinase 1 (TAK1) at the Crossroad of B Cell Receptor and Toll-Like Receptor 9 Signaling Pathways in Human B Cells

**DOI:** 10.1371/journal.pone.0096381

**Published:** 2014-05-06

**Authors:** Dániel Szili, Zsuzsanna Bankó, Eszter Angéla Tóth, György Nagy, Bernadette Rojkovich, Tamás Gáti, Melinda Simon, Zoltán Hérincs, Gabriella Sármay

**Affiliations:** 1 Department of Immunology, Eötvös Loránd University, Budapest, Hungary; 2 Buda Hospital of Hospitaller Brothers of St. John, Budapest, Hungary; 3 Department of Rheumatology, Semmelweis University, Budapest, Hungary; University of Miami, United States of America

## Abstract

B cell development and activation are regulated by combined signals mediated by the B cell receptor (BCR), receptors for the B-cell activating factor of the tumor necrosis factor family (BAFF-R) and the innate receptor, Toll-like receptor 9 (TLR9). However, the underlying mechanisms by which these signals cooperate in human B cells remain unclear. Our aim was to elucidate the key signaling molecules at the crossroads of BCR, BAFF-R and TLR9 mediated pathways and to follow the functional consequences of costimulation.Therefore we stimulated purified human B cells by combinations of anti-Ig, B-cell activating factor of the tumor necrosis factor family (BAFF) and the TLR9 agonist, CpG oligodeoxynucleotide. Phosphorylation status of various signaling molecules, B cell proliferation, cytokine secretion, plasma blast generation and the frequency of IgG producing cells were investigated. We have found that BCR induced signals cooperate with BAFF-R- and TLR9-mediated signals at different levels of cell activation. BCR and BAFF- as well as TLR9 and BAFF-mediated signals cooperate at NFκB activation, while BCR and TLR9 synergistically costimulate mitogen activated protein kinases (MAPKs), ERK, JNK and p38. We show here for the first time that the MAP3K7 (TGF beta activated kinase, TAK1) is responsible for the synergistic costimulation of B cells by BCR and TLR9, resulting in an enhanced cell proliferation, plasma blast generation, cytokine and antibody production. Specific inhibitor of TAK1 as well as knocking down TAK1 by siRNA abrogates the synergistic signals. We conclude that TAK1 is a key regulator of receptor crosstalk between BCR and TLR9, thus plays a critical role in B cell development and activation.

## Introduction

B cell receptors (BCR) play a central role in B cell development, activation, survival and cell death [1], [2]. B cell's fate is determined by the strength of signals mediated by BCR and a plethora of other receptors, including the innate receptor, TLR9 and the receptors of B cell activating factor of the tumor necrosis factor family (BAFF-R) [3]–[6]. Modulation of BCR induced pathways upon ligand binding to BAFF-R and TLR9 modifies the strength of the signal that may lead to an aberrant response, consequently, survival and activation of autoreactive B cells [7]–[10].

BAFF is the ligand for three TNF family receptors, namely BAFF-R (or BR3), transmembrane activator, calcium modulator, cyclophilin ligand interactor (TACI) and B cell maturation antigen (BCMA), but only its interaction with BAFF-R is indispensable for B cell survival [11], [12]. BCMA is not expressed on resting B cells; furthermore, BAFF exerts its survival effect on TACI deficient cells as well. All of these data indicates that BAFF-R is the dominant receptor that mediates BAFF-dependent effects to B cells [13]. BAFF mediated signals are necessary for the normal B cell development. In absence of BAFF mature B cells do not develop, and in the contrary, increased level of BAFF may result in survival of autoreactive cells that escape from the negative selection [14]–[16]. An elevated level of BAFF was detected in sera of Systemic lupus erythematosus (SLE) patients [17]. Thus BCR and BAFF cosignaling may potentiate the risk for autoimmunity. Inhibitor κB kinase 1 (IKK1) serves as a major coordinator of signal transduction downstream of BAFF-R that regulates BAFF-induced B cell survival and growth. BAFF induces multiple signaling pathways, and activates NFκB both on the classical and on an alternative way that requires IKK1 expression and promotes p100 processing to p52 [5], [18]. BAFF-induced AKT activation increases the metabolic fitness of B cells, while sustained ERK1/2 activation leads to phosphorylation of the pro-apoptotic Bcl-2 family member Bim [10], [19], [20]. BAFF also activates c-Jun N-terminal (JNK) and p38 MAPKs in human B cells that have role in activation induced cytidine deaminase (AID) expression and class switch recombination [21], [22].

Stimulation of B cells via BCR triggers various signaling events. First the tyrosine phosphorylation cascade is activated that results in the recruitment of protein kinase C-β (PKCβ) to the cell membrane, which in turn triggers the formation of a 3-component complex composed of the CARD domain proteins, CARMA1, BCL10 and MALT1 [23], [24]. The formation of this ternary complex leads to the activation of the IKK complex through recruiting the ubiquitin E3 ligase TRAF6, resulting in the ubiquitination of TRAF6 itself and IKKγ [25]. In turn, transforming growth factor-β–activated kinase 1 (TAK1) is activated, which then phosphorylates and activates IKKβ [26]. TAK1 also activates the members of the mitogen activated protein kinase family (MKK family), which in turn phosphorylate and activate JNK and p38 kinases [27].

The innate receptor TLR9 is essential for recognition of microbial hypomethylated CpG-DNA or its analog, synthetic oligodeoxynucleotide, enriched of CpG motifs (CpG-ODNs). All TLRs share a similar cytosolic domain termed the Toll-IL-1R (TIR) domain that recruits other TIR domain-containing adaptors such as myeloid differentiation primary gene 88 (MyD88) [28]. In turn, MyD88 recruits IRAK1 (IL-1 Receptor-associated kinase) and IRAK4. IRAK1 binds to TRAF6, which then catalyzes K63 polyubiquitination, leading to the activation of a TAK1 [26]. Dual BCR and TLR signals may potentiate the risk for autoimmunity. Although B cell development and survival is not modified in the absence of TLR signals, patients with MyD88 or IRAK4 deficiency have an altered BCR repertoire with an increased proportion of autoreactive B cells, due to the impaired B cell selection processes [29]. Others have shown that TAK1 is critical for B-cell maturation and BCR-induced NF-κB activation [30].

TAK1 is involved in various signaling pathways contributing to cell activation, development, survival and function, therefore may play a critical role in connecting innate and adaptive immunity [31]–[33]. Numerous studies have shown that elevated activating signals or decreased inhibitory potential can lead to the development of autoreactive B cells [34]–[37]. It is also known that elevated level of BAFF, or dual BCR and TLR signals may potentiate the risk for autoimmunity [12], [38]. We have recently reported that both BAFF-R and TLR9 can collaborate with BCR to protect B-cells from Fas-induced programmed death [39].

In this study we aimed to investigate the effect of the combined stimulation of human B cells via BCR, TLR9 and BAFF-R on a variety of early and late signaling events. We have shown for the first time that TAK1 is in a prominent position at the crossroads of the BCR and TLR9 stimulated signaling pathways, while BAFF collaborate with anti-Ig or TLR9 initiated signals by contributing to NFκB activation.

## Materials and Methods

### Ethic statement

Blood samples were taken from healthy blood donors and from patients suffering in Rheumatoid arthritis after written consent with ethical permission of the Scientific Research Ethics Committee of the Medical Scientific Board of Ministry of Human Resources (ETT TUKEB 5257-0/2010-1018EKU 376/PI/010).

Further blood samples were taken from healthy blood donors, and tonsils were obtained from patients undergoing tonsillectomy after written consent with ethical permission of the Scientific Research Ethics Committee of the Medical Scientific Board of Ministry of Human Resources (ETT TUKEB 84-402/2008-1018EKU 1010/PI2008). The Scientific Research Ethics Committee of the Ministry specifically approved these studies.

### Reagents and antibodies

Affinity purified F(ab')_2_ fragment of goat anti-human IgG+IgM (H+L) purchased from Jackson Immunoresearch Laboratories, (London, UK), phosphorothioated unmethylated CpG oligodeoxynucleotide (ODN-2006) (5′-tcgtcgttttgtcgttttgtcgt'-3′) from Sigma Aldrich, (Budapest, Hungary) and recombinant human BAFF, IL-2 and IL-10 obtained from ImmunoTools (Friesoythe, Germany) were used for the stimulation of human B cells. B cell purity was assessed by staining with CD19-APC (BD Biosciences, Franklin Lakes, NJ, USA). Antibodies used in our Western blot experiments, phospho-AKT (Ser473), phospho-ERK (Thr202/Tyr204), phospho-IκB (Ser32), phospho-TAK1 90C7 (Thr184/187), phospho-p38 (Thr180/Tyr182) were purchased from Cell Signaling Technology (Danvers, MA, USA), pFOXO1 (Thr24) and phospho-JNK (Thr183/Tyr185, Thr221/Tyr223) were from Millipore (Billerica, MA, USA). Rabbit anti-SH-PTP1 was used as loading control (Santa Cruz Biotechnology, Santa Cruz, CA, USA). For the phospho-specific flow cytometry PhosFlow Perm Buffer III, CD20-A647 (clone: H1), CD27-PE (clone: L128) and pp38-A488 (clone: 36/p38) (pT180/pY182) were purchased from (BD Biosciences). Secondary goat anti-rabbit IgG-HRP conjugated antibodies were from Cell Signaling Technology (Danvers, MA, USA), Luminata Forte HRP substrate and (5Z)-7-Oxozeaenol (TAK1 inhibitor) were purchased from Millipore (Billerica, MA, USA). Sheep blood was purchased from Bak-Teszt Diagnostical Ltd, Budapest Hungary.

### Isolation and activation of B cells

Burkitt's lymphoma B cell line, BJAB was used for the initial screening of the activation of mitogen-activated protein kinases (MAPK).

Peripheral blood mononuclear cells (PBMCs) were isolated by density gradient centrifugation on Ficoll-Paque PLUS (GE Healthcare, Waukesha, Wisconsin, USA), and B lymphocytes were purified by negative selection using Magnetic Bead-Activated Cell Sorting (MACS) according to the manufacturer's protocol (Miltenyi Biotec, Auburn, CA, USA). B cell purity was assessed by flow cytometry using anti-human CD19-APC antibody. B cells with purity over 95% were used in most experiments, while B cells' purity was over 99% in experiments testing the cytokine production.

Tonsils were obtained from patients undergoing tonsillectomy. Mononuclear cells were isolated by Ficoll-Paque PLUS density gradient centrifugation and T cells were depleted after rosette formation with sheep red blood cells followed by a second density gradient centrifugation. For Western blot analysis, resting tonsil B cells were isolated by Percoll gradient centrifugation [40]. The purity of the resultant B cell suspension was over 95%. Tonsil B cells were further purified by positive magnetic bead separation (Miltenyi Biotech) for the cytokine production experiments.

To analyze synergistic activation, suboptimal doses of stimulants were used. B cells were activated by cross-linking the BCR with 2.5 µg/ml affinity purified F(ab')_2_ fragment of goat anti-human IgG+IgM (H+L) in the presence or absence of 100 ng/ml recombinant human BAFF and 1 µg/ml TLR9 ligand phosphorothioated unmethylated CpG oligonucleotide. The TAK1 inhibitor (5Z)-7-Oxozeaenol was used in 100 nmol. Cell types, different concentrations and times are indicated below the figures.

### Phospho-MAPK Array

For an initial screening of the phosphorylation status of a variety of kinases after BCR and/or TLR9 activation, we have performed a phospho-MAPK array (R&D Systems, Minneapolis, MN, USA). 3×10^6^ BJAB cells were treated with various stimuli per sample for 30 minutes at 37°C. The experiment was carried out according to the manufacturer's instructions.

### Western blot analysis

2×10^6^ peripheral blood or resting tonsil B cells were activated per sample. After 30 minutes of activation, cells were pelleted and lysed in 50 µl lysis buffer (containing 20 mM Tris (pH 7,5), 150 mM NaCl, 1 mM EDTA, 1 mM EGTA, 1% Triton X-100, supplemented with protease and phosphatase inhibitors (2.5 mM Na_3_VO_4_, 1 mM phenyl methyl sulphonyl fluoride

(PMSF), 1.5 mM aprotinin, 10 mM leupeptin). The cell lysates were mixed at 4∶1 with 5 times concentrated reducing sample buffer. Samples were subjected to SDS/PAGE under reduced condition, and proteins were transferred electrophoretically to nitrocellulose membrane (Bio-Rad Laboratories, Hercules CA, USA). The membranes were blocked with 5% BSA in TBS with 0.1% Tween-20 for 1 h, then incubated overnight with the primary antibodies specific for the phosphorylated forms of various signaling proteins. After several washings with Tris-washing buffer (TWB-Tween) the secondary horseradish peroxidase–conjugated species specific antibodies were added, finally, HRPO substrate was added to visualize the indicated molecules by enhanced chemiluminescence (ECL). SH-PTP1 was developed as loading control.

### RNA interference

Small interfering RNA (siRNA) Reagent System and a pool of 4 target-specific siRNA against human TAK1 was purchased from Santa Cruz Biotechnology. BJAB cells were transfected according to the manufacturer's instructions. After 48 and 72 hours cells were activated with 2.5 µg/ml anti-Ig and 1 µg/ml CpG for 30 min and subjected to Western blot analysis.

### Proliferation assay

10^6^–10^7^ purified peripheral blood B cells were incubated with 5 µM carboxyfluorescein diacetate succinimidyl ester (CFSE) in 5% FCS containing PBS for 10 min at 37°C. After repeated washings with ice-cold RPMI 1640 medium, cells were transferred into 96-well plates (4×10^5^ cells/well) and incubated in complete medium with different stimuli for 5 days at 37°C. Cell cultures were harvested and CFSE staining was analyzed by flow cytometry (FACSCalibur, BD Biosciences). Dead cells were stained with propidium iodide (PI) and gated out. The results were evaluated by FlowJo software (Tree Star, Ashland, OR, USA).

### Cytokine production

Secretion of IL-6, IL-8, IL-10 and TNFα cytokines was measured in the supernatants of purified blood and tonsil B cells stimulated under various conditions. 2×10^5^ cells were stimulated per sample. The supernatants were collected after 48 h and stored at −70°C until assayed by Flow Cytomix bead array (Bender MedSystem, San Diego, CA, USA) according to the manufacturers' instruction.

### Plasma blast differentiation

2×10^5^ purified blood B cells were cultured in 96-well plates for 4 days in the presence of IL-2 and IL-10 (50 ng/ml) and different stimuli, and the ratio of CD27^++^, CD38^+^ plasma blasts were evaluated by flow cytometry on FACSAria (BD Biosciences) using CD27-PE and CD38-Alexa Fluor700 antibodies. Dead cells were stained with PI and gated out.

### ELISPOT assay

10^5^ cells added per well of a 96-well round-bottom plate in 200 µl culture media containing IL-2, and IL-10 (50 ng/ml) and different combination of activators as indicated below the figure. Cells were cultured for four days at 37°C and 5% CO_2_.

The frequencies of IgG secreting cells were evaluated by enzyme-linked immunosorbent spot (ELISPOT) assay [41]. Briefly, 96-well MultiScreenHTS-IP Filter Plate (Millipore) were pre-treated with 70% ethanol and washed 3 times in sterile PBS before coated overnight at 4°C with 5 µg/ml mouse anti-human IgG (BD Biosciences). Plates were washed in sterile PBS and blocked with PBS containing 3% BSA. A serial dilution of cultured B cells were added and incubated at 37°C. After 20 h incubation, cells were aspirated and plates were washed with PBS containing 0.1% Tween. Alkaline phosphatase-conjugated mouse anti-human IgG (BD Biosciences) detection antibody (1∶1000) was added, and after two hours of incubation plates were subjected to several washes, then spots were developed with 3-amino-9-ethylcarbazole (AEC, Sigma) solution, and counted using the CTL Immunospot Reader (Cellular Technologies Ltd, Shaker Heights, OH, USA).

### Phospho-kinase specific flow cytometry (Phospho-flow assay)

PBMCs were prepared from peripheral blood of healthy volunteers and of Rheumatoid arthritis (RA) patients having an active disease (DAS28>5,1). Peripheral blood mononuclear cells (PBMC) were separated using Ficoll density gradient centrifugation. 4×10^6^ PBMCs/sample were treated with or without 7.5 µg/ml affinity purified F(ab')_2_ fragment of goat anti-human IgG+IgM (H+L) and 4 µg/ml CpG at 37°C for 30 minutes. After treatment, PBMCs were fixed with 1% paraformaldehyde for 10 minutes at 37°C. The fixed PBMCs were then permeabilized on ice for 30 min using 1 ml Perm Buffer III. Cells were then washed twice and stained with CD20-A647, CD27-PE and pp38-A488. The expression of phosphorylated signaling molecules was analyzed by flow cytometry (FACSCalibur). The amount of phosphorylated p38 MAPK in CD27- naive B cells was presented as mean fluorescence intensity (MFI). The relative expression (%) was calculated based on the change of phosphorylated MAPKs expression in resting and activated cells (stimulated sample's MFI/resting sample's MFI).

### Statistical analysis

Statistical differences of cytokine production and ELISPOT assays were assessed by pairwise comparisons using permutation tests. Briefly, values from the groups to be compared were randomly reassigned to two groups and the difference between the group means was calculated. Distribution of 10000 randomizations was drawn and the two-tailed *P* value corresponding to the real sample assignments was determined. The arithmetic mean of 50 such *P* values was accepted as the probability of α-error. Values of *P*<0.05 were considered significant and were indicated as *, *P*<0.05. Statistical analyses were performed in R (http://www.r-project.org).

The difference in the expression of phosphorylated p38 MAPK between control and activated samples was compared with paired Student's t-test and difference between healthy and RA patients was compared with unpaired Student's t-test (GraphPad Software, San Diego, CA). Values of P<0.05 were considered significant and were indicated as follows: *, P<0.05, **, P<0.01 and ***, P<0.001.

## Results

### Simultaneous activation of B cells via BCR and TLR9 synergistically co-activates MAPKs

The MAPK cascade is a highly conserved module involved in signaling via adaptive and innate receptors, regulating various cellular functions such as cell growth and differentiation.

To find out which kinases are most efficiently costimulated by BCR and TLR9 dual signals, first we performed a human phospho-MAPK protein array using a Burkitt's lymphoma cell line. BJAB cells were stimulated by suboptimal doses of anti-Ig and CpG ODN for 30 min then lysed and samples were tested for phosphorylated kinases.

**Figure 1:**
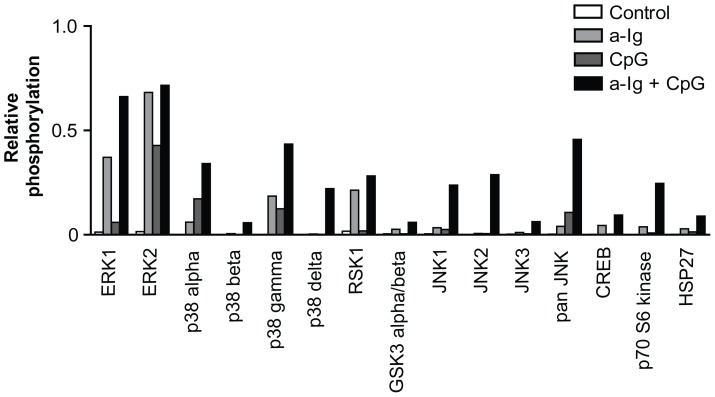
Screening of BCR and TLR9 stimulated kinase activation pattern in BJAB Burkitt's lymphoma cells by MAPK protein profiler array. BJAB cells were treated with 5 µg/ml anti-Ig and/or 2 µg/ml CpG for 30 minutes. Relative phosphorylation values were calculated as the ratios of signal strengths for each kinases as compared to positive control spots. Only kinases that were activated are shown.

The phospho-MAPK array kit can differentiate 24 kinases, and only those that were costimulated by anti-Ig and CpG ODN are shown. In this screening experiment we identified ERK1, various isoforms of p38 and JNK exhibiting synergistically elevated phosphorylation level after suboptimal double stimulation of BJAB cells through BCR and TLR9 as compared to unstimulated control cells (Fig. 1). The highest degree of synergism was observed in activation of JNK, but we could not detect any synergism in AKT phosphorylation.

### BCR and TLR9 synergistically activates TAK1 and its downstream target p38 MAPK

**Figure 2:**
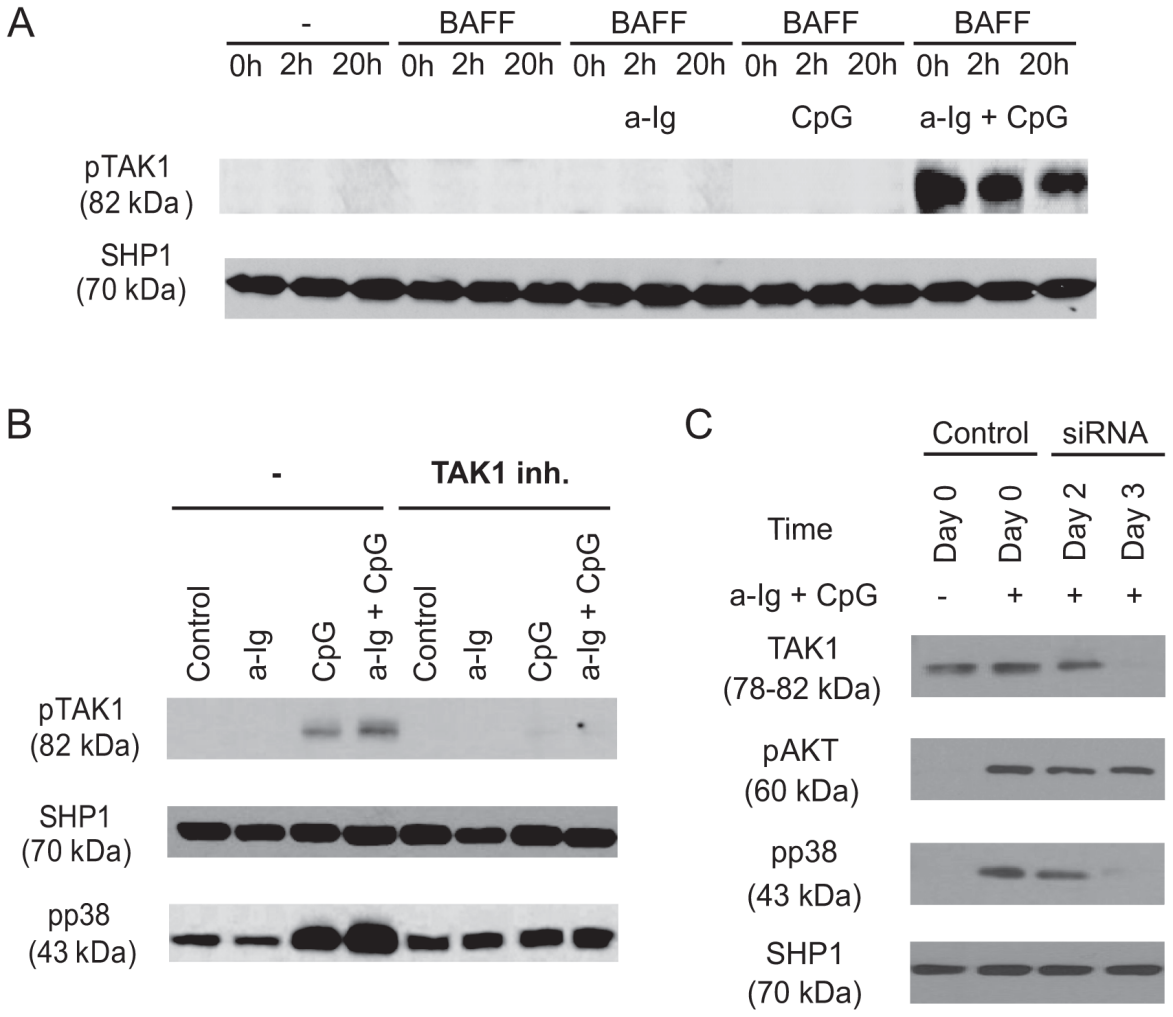
BCR and TLR9 induced signals synergistically activate TAK1 and p38 MAPK in human B cells that is independent of BAFF. (A) Purified human B cells were left untreated (0 h) or pretreated with 100 ng/ml BAFF for 2 h or 20 h, and then in the last 30 min of pretreatment were activated with anti-Ig (2.5 µg/ml) and/or CpG (1 µg/ml), (B) B cells were stimulated with anti-Ig (2.5 µg/ml), CpG-ODN (2 µg/ml) or the combination of both reagents as indicated for 30 min, in the absence (-) or presence of specific TAK1 inhibitor, (5Z)-7-Oxozeaenol, then samples were subjected to Western blot analysis using pTAK1 or pp38 MAPK specific antibodies. C) Control and TAK1-specific siRNA transfected BJAB cells were activated with 2.5 µg/ml anti-Ig and 1 µg/ml CpG for 30 minutes, and subjected to Western blot analysis to measure TAK1, pAKT and pp38 level. SHP1 was used as a loading control.

After the initial screening phospho-MAPK on BJAB cell line we turned to primary B cells isolated from peripheral blood of healthy donors. One of the upstream regulators of ERK, JNK and p38 MAPK is the MAP kinase kinase kinase 7 (MAP3K7), TAK1, and it was previously shown that BCR, TLR9 and TACI mediated signals activate TAK1 [3], [29], [30]. Therefore we checked whether the two or three suboptimal stimuli are synergizing at the level of TAK1 phosphorylation in human B cells. Peripheral blood B cells were stimulated for 30 min by suboptimal doses of anti-Ig and/or CpG ODN, in the presence or absence of stimulation by BAFF for different time intervals, and TAK1 phosphorylation was tested by Western blot. To assess the effect of BAFF, cells were incubated in the presence of BAFF for two or twenty hours respectively, since different optimal activating time periods for BAFF were reported earlier [19]. Suboptimal BCR or TLR9 stimulation with or without BAFF did not provoke the phosphorylation of TAK1, while the combined BCR and TLR9 stimuli were capable to induce a strong synergistic TAK1 phosphorylation that was independent of BAFF (Fig. 2A).

One of the downstream targets of TAK1 is p38 MAPK, therefore we monitored the phosphorylation of p38 upon the same stimuli. Although the basal phosphorylation of p38 MAPK was relatively high, suboptimal activation of B cells via BCR and TLR9 was sufficient to induce - in parallel with TAK1 – a synergistic p38 phosphorylation (Fig. 2B).

To test the TAK1 dependency of the synergistic effect, we applied a highly specific inhibitor of TAK1, (5Z)-7-Oxozeaenol. This inhibitor abolished TAK1 phosphorylation, as expected, and blocked p38 phosphorylation induced by the single (anti-Ig or CpG ODN) as well as by double stimuli, indicating that the synergistic activation of p38 by BCR and TLR9 stimuli is TAK1 dependent (Fig. 2B).

Next we examined the molecular and functional consequences of TAK1 gene silencing in BJAB Burkitt's lymphoma cells. Using a pool of 4 TAK1-specific siRNA, we found that TAK1 was effectively silenced after 72 h. To further investigate the TAK1 dependency of the synergistic p38 activation in anti-Ig and CpG ODN costimulated cells, p38 phosphorylation was compared in TAK1 siRNA transfected and untransfected samples. As shown in Fig. 2C, the double stimuli induced AKT phosphorylation in both the untransfected and the siRNA transfected BJAB cells, while p38 phosphorylation was completely blocked in the latter TAK1 knocked down sample. These data suggest that TAK1 is responsible for the synergistic activation of B cells by BCR and TLR9.

### Inhibition of TAK1 impairs the BCR and TLR9 induced coactivation of MAPKs, but not that of AKT

**Figure 3:**
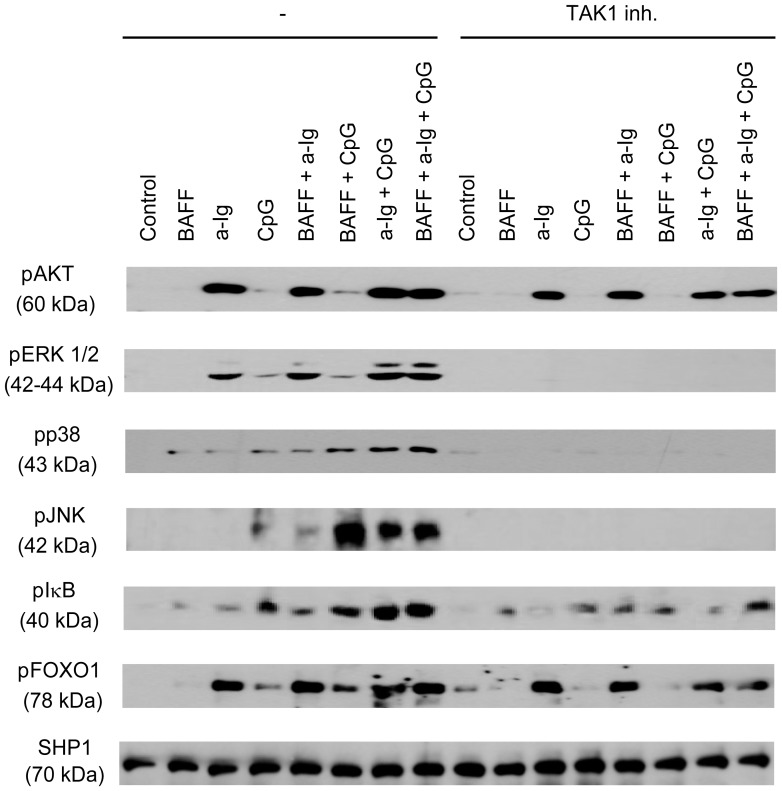
TAK1 dependent activation of the MAPK and NFκB pathways in B cells stimulated with combinations of anti-IgG, CPG ODN and BAFF. Resting tonsill B cells were stimulated with combinations of anti-Ig (2.5 µg/ml), BAFF (100 ng/ml) and CpG (2 µg/ml) for 30 min with or without TAK1 inhibitor ((5Z)-7-Oxozeaenol), and the phosphorylation of various signaling molecules was tested by Western blot.

To examine whether the simultaneous suboptimal stimuli through BCR, TLR9 and BAFF-R result in a significant B cell activation, first we monitored the phosphorylation of different signaling molecules following the single, double or triple stimuli (Fig. 3).

30 min treatment with BAFF slightly induced phosphorylation of p38, and IκB, but alone it was a weak activator. However, it enhanced CpG ODN induced phosphorylation of p38, JNK and IκB, but not the other signaling molecules tested. These data indicate that BAFF cooperates not only with the BCR triggered but also with TLR9 induced signals.

30 minutes suboptimal activation by anti-IgG/M induced a strong AKT, ERK and FOXO1 phosphorylation, and weak p38 and IκB responses that were remarkably enhanced by both CpG and BAFF. On the other hand, anti-Ig induced a visible JNK phosphorylation only in the presence of BAFF or CpG ODN.

1 µg/ml CpG alone induced slight, but together with anti-Ig a strong phosphorylation of ERK, p38, JNK and IκB, confirming the results of the phospho-MAPK array. While simultaneous BCR and TLR9 stimulation showed a strong synergy, addition of BAFF could not enhance further the phosphorylation level of critical signaling molecules in the triple stimulated cells. AKT kinase and its downstream target, FOXO1 transcription factor was dominantly activated by anti-Ig, and was not influenced by BAFF and CpG ODN.

Preincubation of cells with specific TAK1 inhibitor before the various stimuli were added resulted in a decreased pIκB level, while ERK, p38 and JNK phosphorylation was totally abolished. As pAKT was not affected by TAK1 inhibition, FOXO1 also has shown the same phosphorylation profile with or without inhibitor.

### BCR and TLR9 mediated simultaneous, suboptimal stimuli enhance B cell proliferation that is partially blocked by TAK1 inhibition

**Figure 4:**
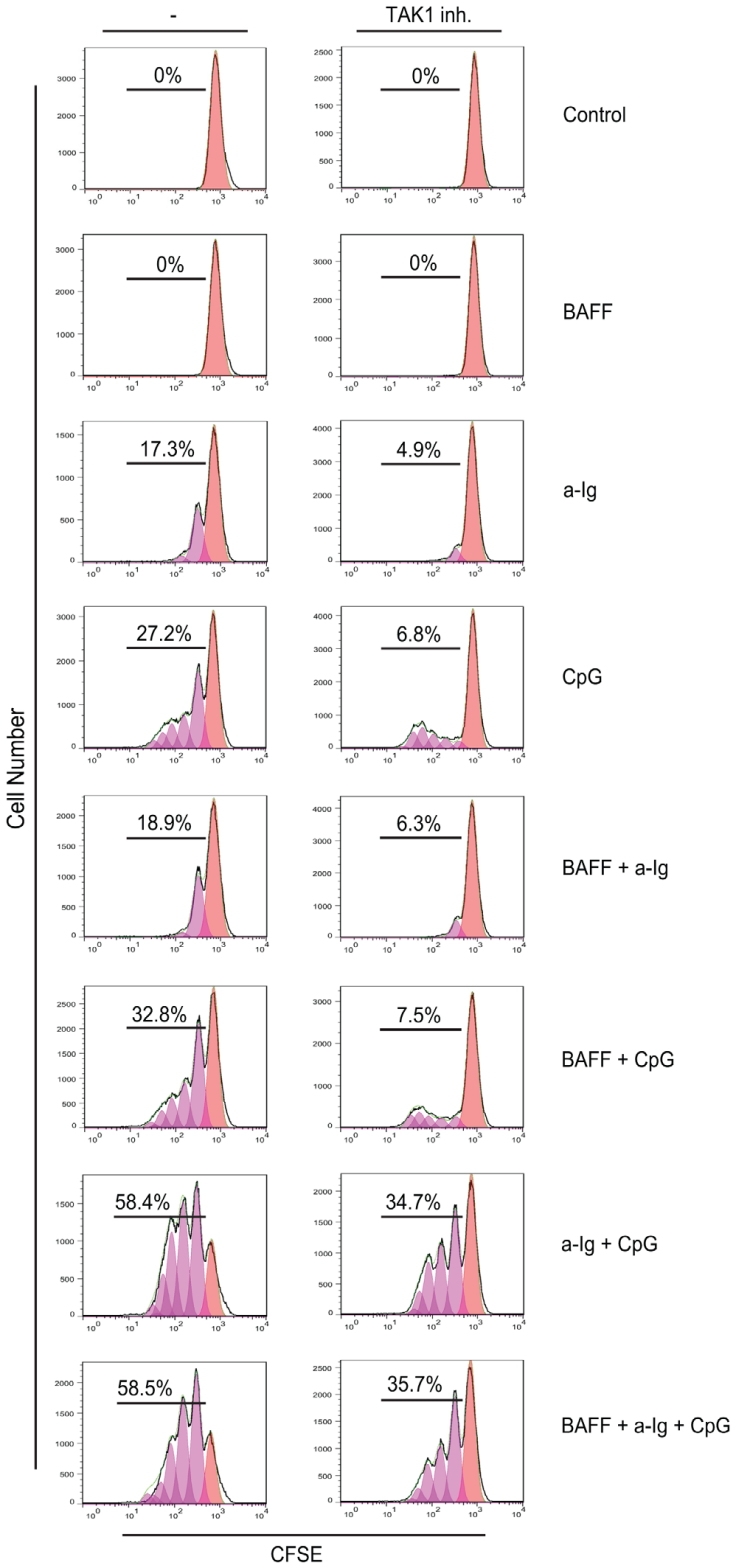
Proliferation of primary human B cells after the combined stimulation with anti-Ig, CPG ODN and BAFF. Representative flow cytometric histograms of CFSE-labeled blood human B cells stimulated with combinations of 2.5 µg\ml anti-Ig, 100 ng/ml BAFF and 2 µg/ml CpG as indicated. Peaks shifted to the left represent cell populations undergoing increasing number of division. Total percentages of dividing cells are shown. The samples were cultured in the presence or absence of TAK1 inhibitor.

Since we observed a synergistic co-activation at the level of various signaling molecules, next we investigated the effect of BCR, BAFF-R and TLR9 mediated signals on B cell proliferation. It is well known that both antigen mimicking stimuli and the TLR9 ligand CpG ODN induce cell proliferation, while BAFF - through the regulation of gene expression - contributes to B cell survival [13]. As an indicator of cell proliferation, fluorescence intensities of CFSE loaded cells were tested after 5 days culture in the presence of various stimuli. BAFF did not induce proliferation but slightly enhanced proliferation when it was added to BCR or TLR9 stimulated samples, while had no/little effect on proliferation triggered by BCR and TLR9 mediated dual signals (Fig. 4). In the presence of TAK1 inhibitor the proliferation rate was lower in all samples, however the synergism between BCR and TLR9 was still observed. Thus we conclude that simultaneous suboptimal stimuli of anti-Ig and CpG results in a robust TAK1 dependent B cell expansion.

### The role of TAK1 in cytokine secretion of B cells upon BCR and/or TLR9 stimulation

**Figure 5:**
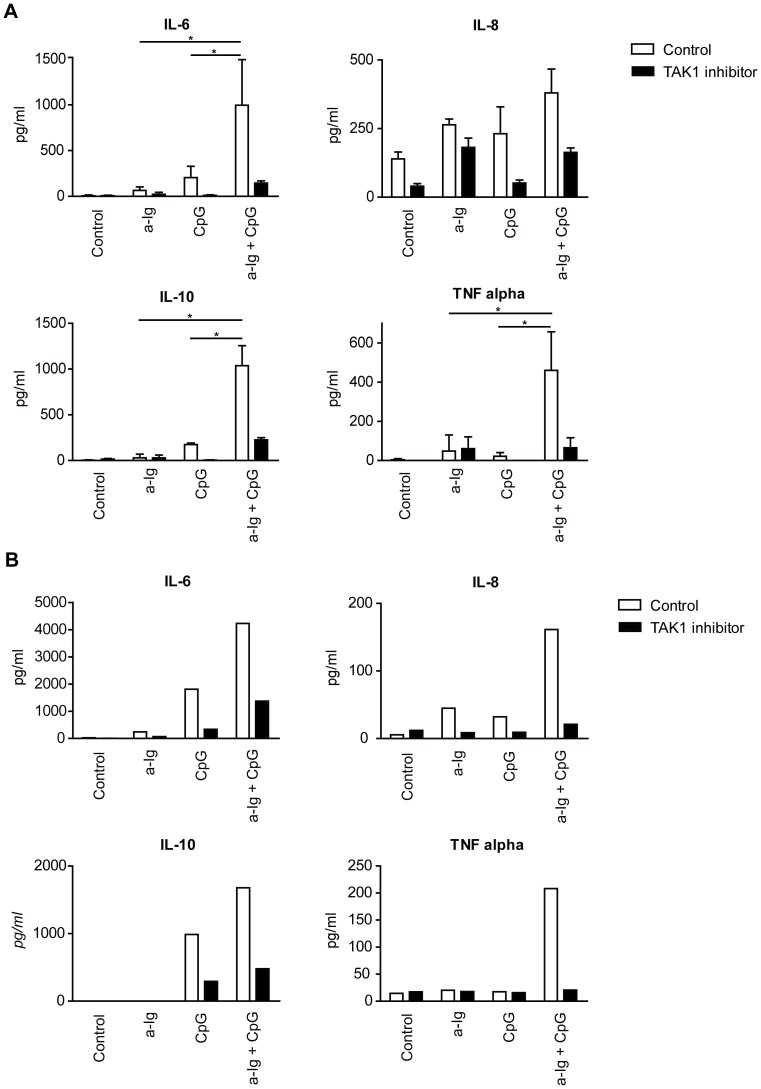
BCR synergizes with TLR9 on a TAK1 dependent manner for cytokine production in primary human B cells. Secreted IL-6, IL-8, IL-10 and TNFα were measured after 48 h from culture supernatants of vehicle treated (white bars) and TAK1 inhibitor treated (black bars) purified human tonsill (A) and blood (B) B cells. Data represent the mean ±SD of three different experiments with resting tonsill B cells (A), while one experiment with blood B cells (B) is shown. *p<0.05.

Human B cells produce both pro- and anti-inflammatory cytokines in response to various stimuli [42], [43]. BCR and TLR9 costimulation may enhance IL-6, IL-10 and TNFα secretion [44]. The role of TAK1 on cytokine synthesis was demonstrated in TAK1 deficient mice and by TAK1 gene silencing experiments [33], [45]. We addressed the question whether the combined suboptimal anti-Ig, CpG and BAFF stimuli induce a synergistic cytokine production by B cells. Dual BCR and TLR9 stimulation have a synergistic effect on IL-6, IL-8, IL-10 and TNFα secretion of resting human tonsil (Fig. 5A) and blood B cells (Fig. 5B), which was diminished by the TAK1 inhibitor (Fig. 5). BAFF had no effect when used alone and had an ambiguous, marginal effect when it was combined with anti-Ig and/or CpG stimuli (data not shown).

### The enhanced plasma blast (PB) generation and IgG secretion of human B cells costimulated by anti-Ig and CPG ODN is decreased by the inhibition of TAK1

**Figure 6:**
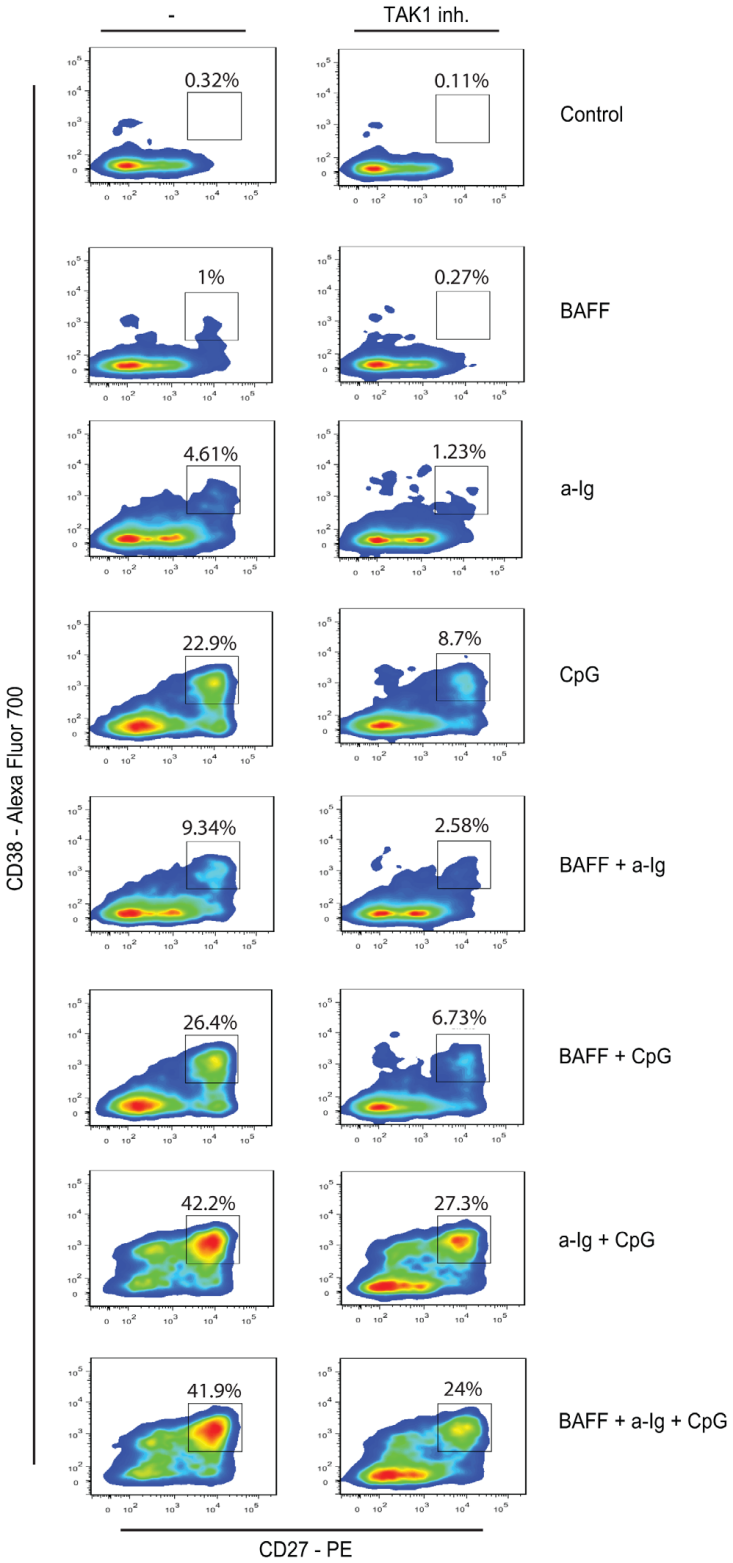
Plasma blast generation induced by single or combined stimuli of B cells via BCR, TLR9 and BAFF-R is partially inhibited by (5Z)-7-Oxozeaenol. Purified B cells (2×10^5^ cells/well) were cultured with anti-Ig (2.5 µg/ml), BAFF (100 ng/ml) and CpG (0.5 µg/ml) for 4 days in the presence of IL-2 (50 ng/ml) and IL-10 (50 ng/ml). Percentages of CD27^++^ CD38^+^ plasma blast cells were evaluated by flow cytometry. Samples in the right column were treated with 5Z-7 Oxozeaenol (100 nmol).

Stimulation of B cells via BCR, TLR9 and BAFF-R is capable to induce plasma blast differentiation [46]–[48]. We have investigated whether TAK1 inhibitor is able to influence BCR and TLR9 induced plasma cell differentiation and Ig secretion. B cells were incubated in the presence of anti-Ig, CpG ODN, IL-2 and IL10. Cells were defined as: CD27^−^ CD38^−^ naive B cells, CD27^+^ CD38^−^ memory B cells, CD27^−^ CD38^+^ activated naive cells and CD27^++^ CD38^+^ plasma blasts. The highest CD27^++^ CD38^+^ plasma blast ratio was observed in the anti-Ig and CpG ODN double stimulated samples (Fig. 6). While BAFF didn't contribute to plasma blast generation in the triple stimulated samples, it successfully elevated plasma blast ratio when added either to anti-Ig or to CpG ODN stimulated cells. Inhibition of TAK1 decreased the ratio of CD27^++^ CD38^+^ plasma blasts in each sample, while the trend of synergism was still noticeable.

**Figure 7:**
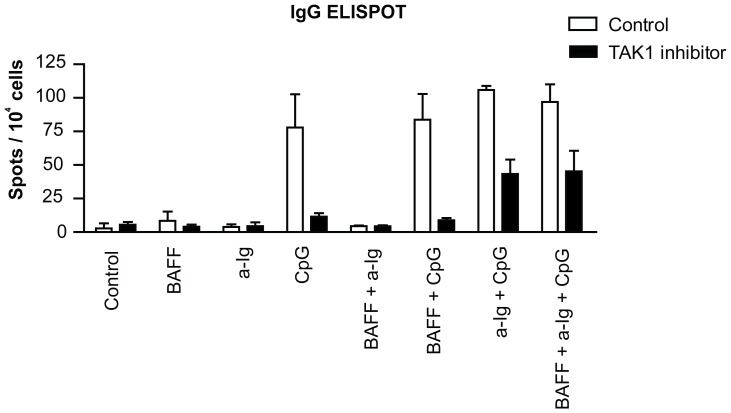
Synergistic stimulation of IgG producing plasma cell differentiation by anti-Ig and CpG ODN in the presence of TAK1 inhibitor. Purified human blood B cells (10^5^ cells/well) were cultured with anti-Ig (2.5 µg/ml), BAFF (100 ng/ml) and CpG (0.5 µg/ml) as indicated below the figure for 4 days in the presence of IL-2 (50 ng/ml) and IL-10 (50 ng/ml). Numbers of IgG producing plasma cells were determined by ELISPOT assay. Spot numbers in the presence of vehicle (white bars) and spots in the TAK1 inhibitor treated samples (black bars) are shown. Means ±SD of three different experiments.

It was shown by Sato and others that IgG production was impaired in B cell-specific TAK1 deficient mice [33]. To elucidate the effect of TAK1 dependent costimulation on antibody production of purified human B cells, we have measured the frequency of IgG producing cells after 4 days culture in the presence of anti-Ig and CpG ODN by ELISPOT assay (Fig. 7). Only CpG ODN stimuli induced IgG production alone. When anti-Ig was added to CpG ODN we observed a slight, not significant increment, while BAFF did not contribute to any of the combinations. As expected, TAK1 inhibition decreased the number of IgG producing plasma cells. Interestingly, in the presence of (5Z)-7-Oxozeaenol both BCR and TLR9 signals alone were insufficient to trigger IgG production, while dual stimulation by anti-Ig and CpG ODN elevated the number of IgG producing cells as compared to the single stimulated samples, indicating that the synergistic signals partly overcome the effect of the inhibitor.

### Phospho-flow analysis of p38 MAPK activation in B cells of RA patients and healthy individuals

**Figure 8:**
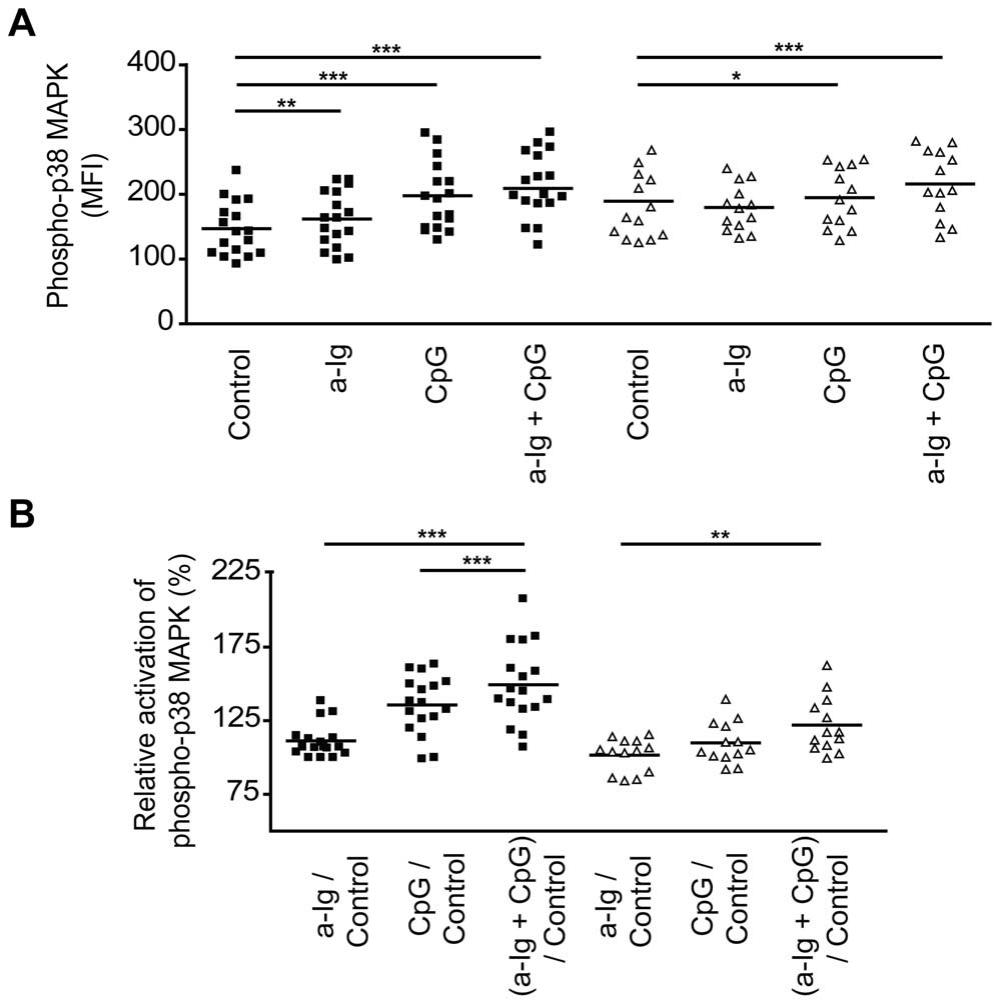
Phospho-flow analysis of p38 MAPK activation in resting and activated B cells from healthy blood donors and from RA patients. PBMC (4×10^6^ cells) were left unstimulated (control) or stimulated with anti-Ig (7.5 µg/ml), CpG (4 µg/ml) or both agents for 30 min at 37°C. p38 phosphorylation was tested in the CD19^+^ CD27^−^ naive B cell subset. (A) The activation of p38 MAPK in healthy (square; *n* = 17) and RA (triangle; *n* = 13) B cells were analyzed by flow cytometry. (B) The relative activation of p38 MAPK was calculated based on the alteration of phosphorylated MAPKs expression in resting and stimulated naive B cells (stimulated sample MFI/resting sample MFI). Paired Student's t-test was used to assess the differences of phospho-p38 MAPK expression between control and stimulated samples. *p<0.05, **p<0.005, ***p<0.0005.

Since costimulation of B cells via BCR and TLR9 was shown to break tolerance and induce autoimmunity, we were interested to study the effect of dual BCR and TLR9 signals on MAPK activation in B cells of RA patients. We monitored phospho-p38 MAPK since it has shown a pronounced synergistic TAK1 dependent coactivation by BCR and TLR9 stimuli in healthy B cells. Because of the limited amount of blood we could obtain from the patients, we applied phospho-flow technique, and compared the level of p38 MAPK phosphorylation in CD19^+^ CD27^−^ naïve B cells from healthy blood donors and RA patients. We confirmed the results of Western blot experiments; BCR and TLR9 double signals induced a significantly higher p38 MAPK phosphorylation as compared to cells stimulated with the single signals (Fig. 8A). However, since the basal level of p38 MAPK phosphorylation was higher in RA patients as compared to healthy control, the relative phosphorylation of p38 MAPK in the single or double stimulated samples was significantly lower in RA B cells (anti-Ig: * p = 0.0293; CpG: *** p = 0.0004; anti-Ig+CpG: ** p = 0.040) (Fig. 8 B).

## Discussion

B cell development, growth and differentiation are regulated by signals triggered by ligand binding to a variety of receptors. The innate receptors may directly activate naive B cells in a T cell-independent and antigen-independent manner, therefore the interaction between the innate and adaptive immune systems may play important role in the development of autoimmune diseases and must be strictly regulated to prevent autoimmunity. Beside the antigen specific BCR, B cells express the innate BAFF-R and TLR9 that regulate the survival and activation of B cells. Convergence of the BCR and TLR9 pathway [34], [38], [49]–[51] and also the BCR and BAFF mediated signals [12], [52], [53] were reported previously. It was recently demonstrated that to induce B cell survival BAFF applies BCR and Igα as adaptor proteins in a BAFF-R signaling pathway leading to activation of Syk [53]. Syk-mediated BCR signaling is also a prerequisite for optimal induction of TLR9 [49], furthermore, BTK is essential for co-localisation of the BCR and TLR9 within an auto-phagosome-like compartment and for the synergistic IL-6 production [51]. Additionally, treatment of B cells with soluble or membrane bound BAFF enhances TLR9 expression, thus cosignaling through BAFF-R and TLR9 might have a profound effect on B cells [38], [54]. However, the crosstalk between BAFF-R and TLR9 signaling, furthermore, the molecular mechanism and the functional outcome of the crosstalk between BCR, BAFF-R and TLR9 mediated pathways have not been explored in details.

TLR9 agonist, CpG ODN was used to mimic unmethylated CpG-containing DNA fragments deriving from bacteria or apoptotic cells [55], [56]. We have shown here that BCR and TLR9 costimulation synergistically elevated the phosphorylation level of several isoforms of p38 MAP kinases, JNK and ERK1, while had no effect on other kinases, such as AKT and its downstream kinase, GSK3 (data not shown). We confirmed the initial screening data by other methods such as Western blots and phospho-flow technique as well. These results indicate that there is a high level of synergy between BCR and TLR9 mediated signals in the activation of the MAPK pathway.

TAK1 was originally identified as a mitogen-activated kinase kinase kinase (MAP3K) activated by transforming growth factor-β (TGF-β) [57]. Since then TAK1 was characterized as a central player in multiple immune and inflammatory signaling pathways, including cytokine receptors, TLR, TCR and BCR mediated signaling [24], [30], [31], [33], [58]. TAK1 connects extracellular signals to NFκB activation via the phosphorylation dependent regulation of the inhibitor kB kinase (IKK) complex [31]. Since TAK1 is also involved in the regulation of ERK, JNK and p38 activities, next we tested if TAK1 is phosphorylated by the single or multiple stimuli. We have used suboptimal stimuli allowing us to detect synergy between anti-Ig, CpG ODN and BAFF mediated signals. Although suboptimal BCR or TLR9 signals alone did not induce any visible phosphorylation of TAK1, the dual signals resulted in a robust TAK1 activation, indicating a high level of synergy between the two pathways. However, BAFF did not influence TAK1 phosphorylation in human B cells independently of the time course of treatment. To formally prove that TAK1 is involved in the synergy, a highly specific TAK1 inhibitor, (5Z)-7-oxozeaenol was applied that covalently binds to TAK1 [59]. The inhibitor completely blocked TAK1 phosphorylation induced both by the single and the double BCR and TLR9 signals. The pattern of p38 phosphorylation followed that of TAK1 as expected; synergistic phosphorylation of p38 was also completely blocked by the TAK1 inhibitor. TAK1 silencing with specific siRNA diminished p38 phosphorylation after BCR and TLR9 costimulation, confirming the previous results. TAK1 does not participate in the PI3K pathway regulating AKT, correspondingly, AKT phosphorylation was intact in the same samples, indicating that TAK1 is specifically responsible for the synergistic activation of the MAPK family in B cells costimulated by BCR and TLR9.

In contrast to previous report on mouse B cells [19], [20], we could not detect any effect of BAFF on the activation of ERK, AKT and its substrate FOXO1, however, both molecules were highly phosphorylated by anti-Ig and have shown a weak signal after CpG ODN stimuli. The difference between the mouse and human data might be explained by the species difference and/or the different origin of cells, spleen versus blood, or with the different experimental conditions.

TAK1 was shown to regulate IKK activity by phosphorylating IKKβ at two serine residues within the activation loop, leading to IKK activation [31], [58]. BAFF may also activate IKK complexes on a BTK dependent way, thus contributing to the classical NFκB activation pathway [60]. IKK, by phosphorylating IκB, evokes its ubiquitination and degradation resulting in the activation of NFκB. Testing IκB phosphorylation pattern has revealed that both CpG ODN and BAFF stimulated its phosphorylation and BAFF contributed to both the anti-Ig and the CpG induced effect, however the BAFF induced phosphorylation does not seem to be influenced by TAK1 inhibitor These data suggest that the BAFF stimulated conventional NFκB activation is independent of TAK1.

To see if modulation of intracellular signals by the combined stimuli has any functional significance, next we examined proliferation of CFSE loaded primary B cells treated with single or combined stimuli. As it was expected, BAFF did not influence proliferation, and add very little to the anti-Ig or CpG ODN induced cell proliferation. However, CpG and anti-Ig induced a significant synergistic proliferation of B cells that was partially inhibited by (5Z)-7-oxozeaenol. These data indicate that BCR and TLR9 mediated signals synergistically induce B cell proliferation and that this synergy at least partly depends on TAK1.

In response to various stimuli both mice and human B cells produce cytokines, such as IL-10, IL-6, lymphotoxin α (LT-α) and TNF-α [61]–[63]. Two populations of effector B cells were described (Be1 and Be2) producing different sets of cytokines, while regulatory B cells (Breg) secrete mainly IL-10 [64]–[66]. Therefore we tested if the single or combined signals have any influence on cytokine production of B cells. Human B cells purified from tonsils or blood secreted low levels of IL-6, IL-8, IL-10 and TNFα when they were cultured for 48 h in the presence of either anti-Ig or CpG ODN. However, when both stimuli were added simultaneously, a robust synergistic response was observed in case of IL-6, IL-10 and TNFα that were blocked by TAK1 inhibitor, (5Z)-7-oxozeaenol. IL-1β, IL-12 and IFNγ secretion were not observed (data not shown). These data indicate that the BCR and TLR9 mediated pathways synergistically interact on a TAK1 dependent way to produce both proinflammatory cytokines IL-6 and TNFα and the suppressive cytokine IL-10.

B cells upon activation go through various differentiation stages, finally becoming antibody secreting plasma cells and memory B cells. During this process the cells modulate surface molecules, CD27 appears at the surface of memory cells, while CD27^++^ and CD38^+^ cells are plasma blasts [67]. To see that culturing B cells in the presence of anti-IgG, CpG ODN and BAFF induce plasma blast differentiation we followed the expression of CD27 and CD38 on B cells treated with different stimuli. We have found that BAFF alone had a marginal effect on plasma blast generation, while it doubled the ratio of anti-Ig induced, and somewhat elevated the number of CpG ODN induced plasma blasts. Anti-Ig and CpG ODN once again showed a high level of synergy in inducing the differentiation of plasma blasts, and addition of BAFF had no further effect on this. The partial inhibition by (5Z)-7-oxozeaenol indicates that the synergistic effect depends on TAK1.

Fully differentiated B cells produce antibody, thus to see the effect of co-signaling on this terminal differentiation we tested the number of antibody forming cells in B cell cultures. Only CpG ODN induced significant number of spots that were considerably reduced by (5Z)-7-oxozeaenol. Interestingly, the synergy between BCR and TLR9 induced signals was observed only in the presence of TAK1 inhibitor. These data indicate, that when the CpG induced signal is low (in the presence of inhibitor), the BCR stimulated signals still can act in synergy, stimulating the differentiation of antibody producing plasma cells.

There are evidences in mice and human that TLR9 activation costimulates autoreactive B cells, allows breaking the tolerance and contributes to the pathogenesis of autoimmune diseases [2], [4], [8], [34], [38]. The TLR9 agonist, CpG ODN induces class switching to isotypes IgG2a, IgG2b and IgG3 in mice, while suppressing the production of the IgG1 and IgE isotypes; furthermore, it stimulates class switching from IgM to the more pathogenic IgG1, IgG2 and IgG3 in human [68]–[70]. Recent data demonstrate that 30% of the mature B cells are autoreactive but do not assault self [71]. Receiving signals through TLR9 may activate these dormant autoreactive cells and break tolerance.

Therefore, finally, in order to test if BCR and TLR9 mediated signals act similarly in B cells of healthy individuals and RA patients, the activities of p38 MAPK were compared. The results of phospho-flow experiments confirmed previous data obtained by Western blots, namely, that a significantly elevated p38 phosphorylation has been detected in anti-IgG and CpG ODN co-stimulated healthy samples as compared to the single stimulated cells. On the other hand, although somewhat enhanced level of p38 MAPK phosphorylation was observed in BCR and TLR9 dual stimulated RA B cells, the cells were overall less responsive both to the single and to the double stimuli. We suggest that this relative unresponsiveness is probably due to the high basal phosphorylation rate of p38 in naive B cells. Elevated basal phosphorylation level of MAP kinases in both RA and SLE patients' B cells was observed in previous studies [72], [73]. We hypothesize that B cells from RA patients already received inflammatory signals *in vivo*, therefore they are less responsive to the *in vitro* added ligands.

Taking all together, we conclude that BCR and TLR9 mediated signals collaborate in activating several functions of B cells, such as proliferation, cytokine secretion, generation of plasma blasts and antibody forming plasma cells. BAFF, on the other hand, has a modulating effect on some of these functions. TAK1 plays a crucial role in the synergistic signaling of BCR and TLR9, thus we suggest that TAK1 is a potential drug target in emerging future therapies of autoimmune inflammatory diseases.
