# Expression of Concern: TGFβ Activated Kinase 1 (TAK1) at the Crossroad of B Cell Receptor and Toll-Like Receptor 9 Signaling Pathways in Human B Cells

**DOI:** 10.1371/journal.pone.0265030

**Published:** 2022-03-03

**Authors:** The PLOS ONE Editors

After publication of this article [1], concerns were raised about the pTAK1 panel in Fig 2A: Lanes 1–3 appear similar to lanes 4–6 (rotated 180 degrees) and lanes 7–9. Vertical discontinuities and/or background differences suggestive of splicing were noted after lanes 3, 9, 12.

The authors commented that they had made an error in preparing this panel of Fig 2A by including the same data in lanes 1–3 and 7–9. They further noted that in preparing the figure for publication, they removed some lanes and combined data from two blots. The authors provided an updated version of Fig 2 and underlying data to support the Fig 2A results reported therein (relabelled Fig 2A and 2B in the updated figure). These include the original data for the a-Ig + CpG results, and replacement data for the pTAK1 data in Fig 2A and lanes 1–6 of Fig 2B and the SHP1 data for all lanes in the updated figure. Except for the a-Ig + CpG results, the raw data underlying the pTAK1 and SHP1 results reported in the original Fig 2A were not provided, and so the concerns about the published figure have not been fully resolved. There are artefacts in the pTAK1 (82 kDa): a-Ig BAFF 0h, BAFF 2h, and unstimulated 20h lanes in S1 File that are not present in the original Fig 2A.

In the updated version of Fig 2, the results which were run on separate blots have been separated into two panels (new Fig 2A and 2B); panels C and D in the updated figure are the same as panels B and C in the original figure.

An Academic Editor reviewed the updated figure and replacement data provided and raised concerns that overall question the support for claims of a BAFF-independent synergistic effect of a-Ig+CpG. The Academic Editor noted that pTAK1 levels appear lower in BAFF-treated (2h, 20h) a-Ig+CpG lanes of Fig 2B as compared to the BAFF-untreated (0h) lane; background bands appear different comparing a-Ig, CpG, and a-Ig+CpG lanes on the raw blot image; and Fig 2B and 2C report inconsistent results for the CpG only condition. Of note, the BAFF- (0h) lanes in Fig 2B support a synergistic effect of a-Ig + CpG that does not require co-stimulation with BAFF, but the 2h and 20h BAFF Ig+CpG results suggest BAFF may dampen this effect. Additional data are needed to clarify whether this is a significant and/or reproducible effect. The authors noted that samples used for Fig 2B and 2C were obtained from different blood donors, and that the Fig 2C experiments used twice as much CpG as the Fig 2B experiment. The individual-level differences in lymphocyte basal activation level and/or the different CpG concentration may underlie the different results observed for the CpG only condition. The authors further noted that in Fig 2C the pTAK1 and pp38 panels both support a conclusion that a-Ig+CpG have a synergistic effect, though they acknowledged that the pTAK1 result appears less robust than in Fig 2B.

The authors also clarified that some blot images were stretched for the purpose of presentation in preparing Fig 3. An updated version of Fig 3 is provided here in which the images have not been adjusted in this manner. The raw blots underlying Fig 3 are provided in S4–S9 Files. The authors stated that SHP1 loading controls for Fig 2A (Fig 2A and 2B in the corrected figure) and Fig 3 in [1] were run on separate blots, using the same samples that were used for the pTAK1 experiments.

Due to the nature of the concerns raised about Fig 2 and the unavailability of some data needed to support this figure, the PLOS ONE Editors issue this Expression of Concern.

The authors stated that the original data supporting all other results reported in the article are available upon request.

**Fig 2:**
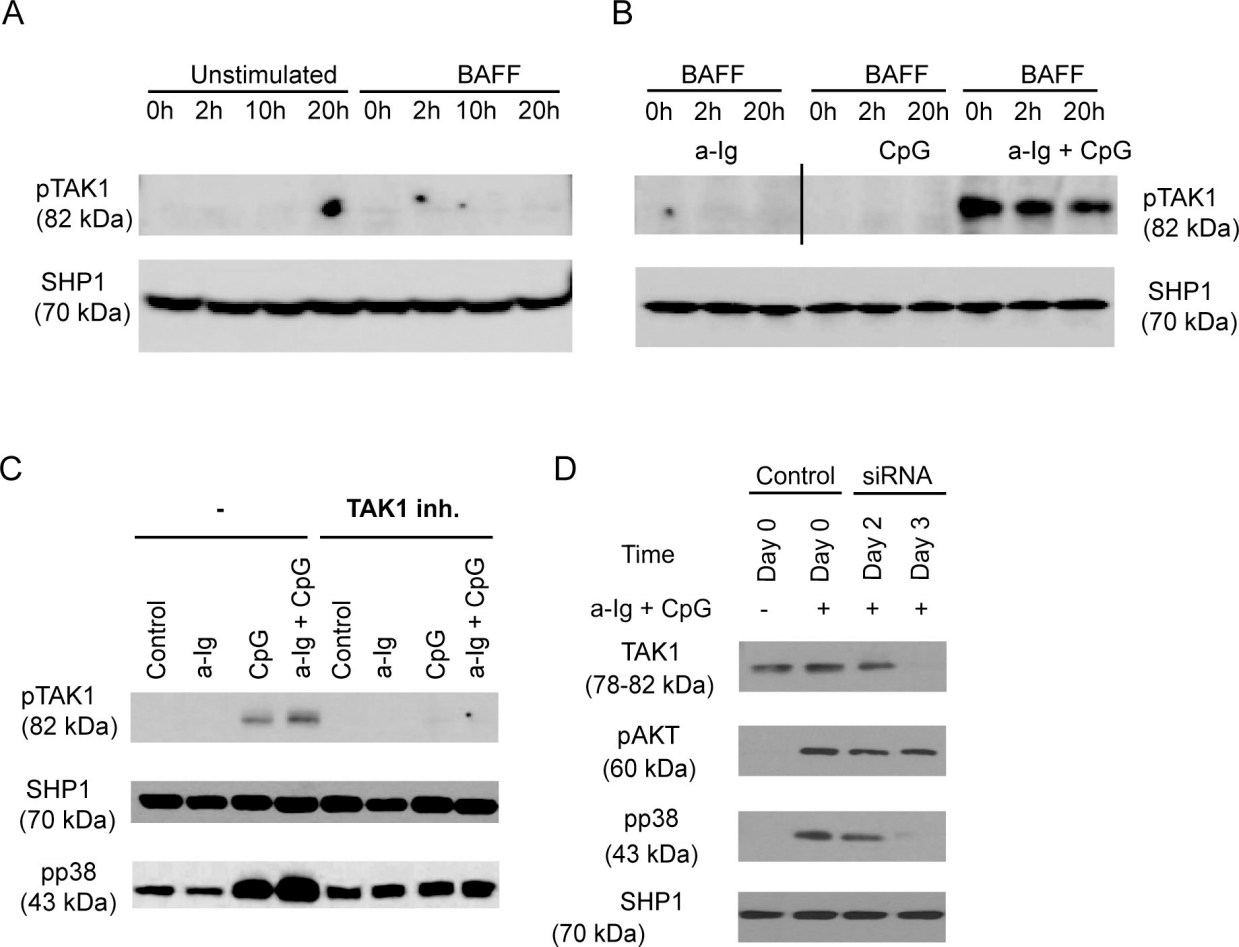
BCR and TLR9 induced signals synergistically activate TAK1 and p38 MAPK in human B cells. (A) Purified human B cells were left untreated (0 h) or pretreated with 100 ng/ml BAFF for 2 h, 10 h or 20 h, (B) BAFF pretreated cells in the last 30 min of pretreatment were activated with anti-Ig (2.5 μg/ml) and/or CpG (1 μg/ml) (black line indicates where membrane was spliced), (C) B cells were stimulated with anti-Ig (2.5 μg/ml), CpG-ODN (2 μg/ml) or the combination of both reagents as indicated for 30 min, in the absence (-) or presence of specific TAK1 inhibitor, (5Z)-7-Oxozeaenol, then samples were subjected to Western blot analysis using pTAK1 or pp38 MAPK specific antibodies. (D) Control and TAK1-specific siRNA transfected BJAB cells were activated with 2.5 μg/ml anti-Ig and 1 μg/ml CpG for 30 minutes, and subjected to Western blot analysis to measure TAK1, pAKT and pp38 level. SHP1 was used as a loading control.

**Fig 3:**
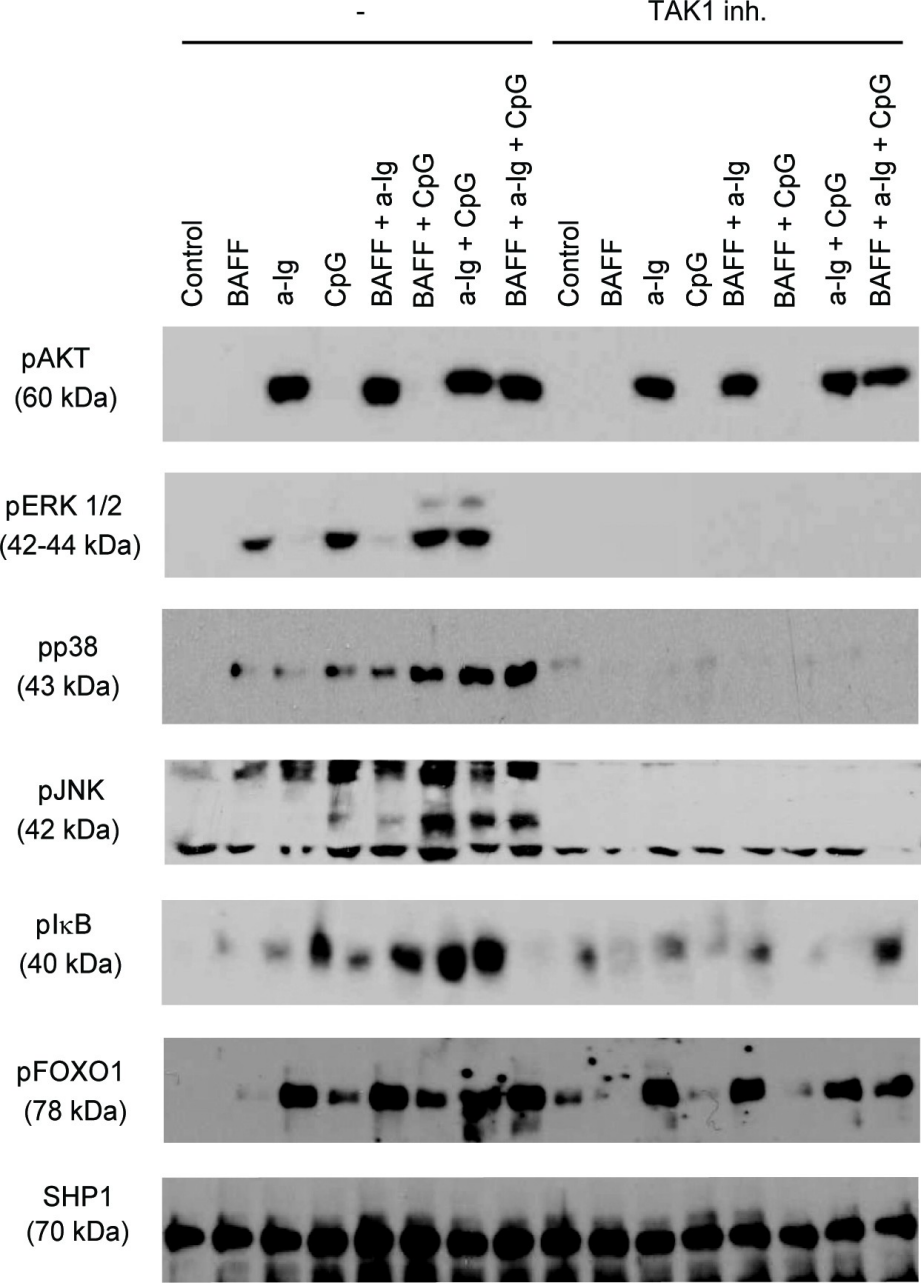
TAK1 dependent activation of the MAPK and NFκB pathways in B cells stimulated with combinations of anti-IgG, CPG ODN and BAFF. Resting tonsill B cells were stimulated with combinations of anti-Ig (2.5 μg/ml), BAFF (100 ng/ml) and CpG (2 μg/ml) for 30 min with or without TAK1 inhibitor ((5Z)-7-Oxozeaenol), and the phosphorylation of various signaling molecules was tested by Western blot.

## Supporting information

S1 File Original blot data supporting pTAK1 results in corrected Fig 2A and 2B. (PDF)

S2 File Original blot data supporting SHP1 results in corrected Fig 2A. (PDF)

S3 File Original blot data supporting SHP1 results in corrected Fig 2B. (PDF)

S4 File Original blot data supporting pAkt and pERK results in Fig 3. (PDF)

S5 File Original blot data supporting pp38 results in Fig 3. (PDF)

S6 File Original blot data supporting pJNK results in Fig 3. (PDF)

S7 File Original blot data supporting pIkB results in Fig 3. (PDF)

S8 File Original blot data supporting pFOXO1 results in Fig 3. (PDF)

S9 File Original blot data supporting SHP1 results in Fig 3. (PDF)

## References

[1] Szili D, Bankó Z, Tóth EA, Nagy G, Rojkovich B, Gáti T, et al. (2014) TGFβ Activated Kinase 1 (TAK1) at the Crossroad of B Cell Receptor and Toll-Like Receptor 9 Signaling Pathways in Human B Cells. PLoS ONE 9(5): e96381. 10.1371/journal.pone.0096381 24801688 PMC4011794

