# Supplementary figures and images for: Expression of Concern: TGFβ Activated Kinase 1 (TAK1) at the Crossroad of B Cell Receptor and Toll-Like Receptor 9 Signaling Pathways in Human B Cells
Source: PLoS One. 2022 Mar 3;17(3):e0265030. doi: 10.1371/journal.pone.0265030 (PMC8893696; doi:10.1371/journal.pone.0265030)

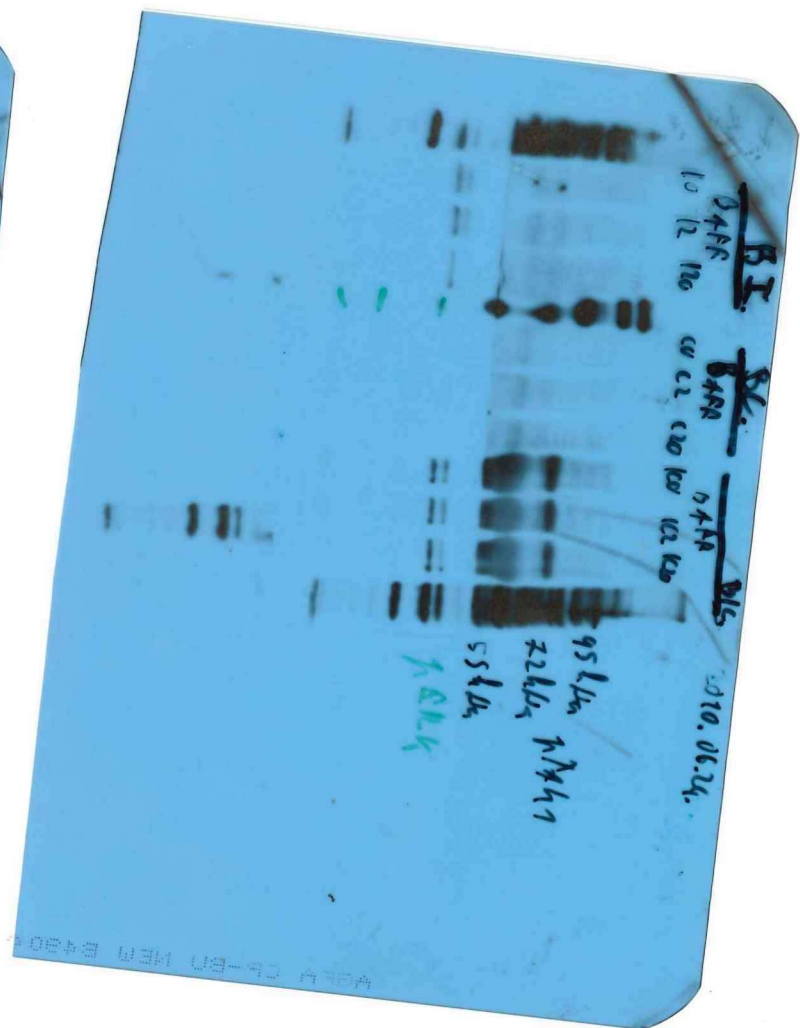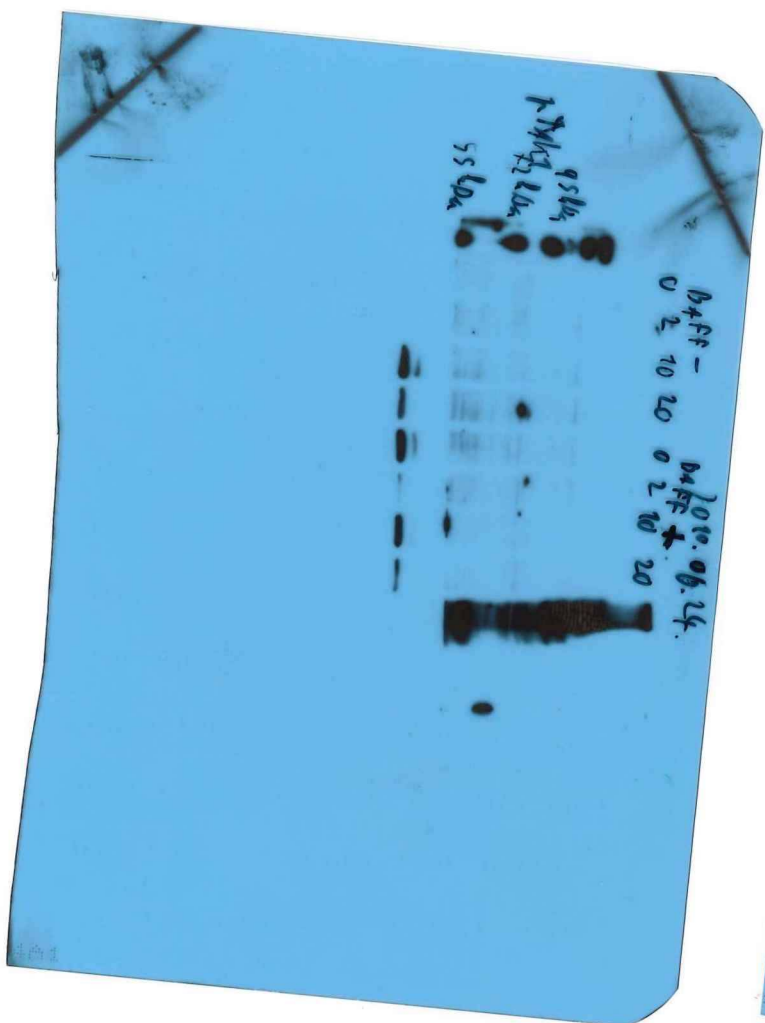

Supplement: S1 File — (PDF) [file pone.0265030.s001.pdf]

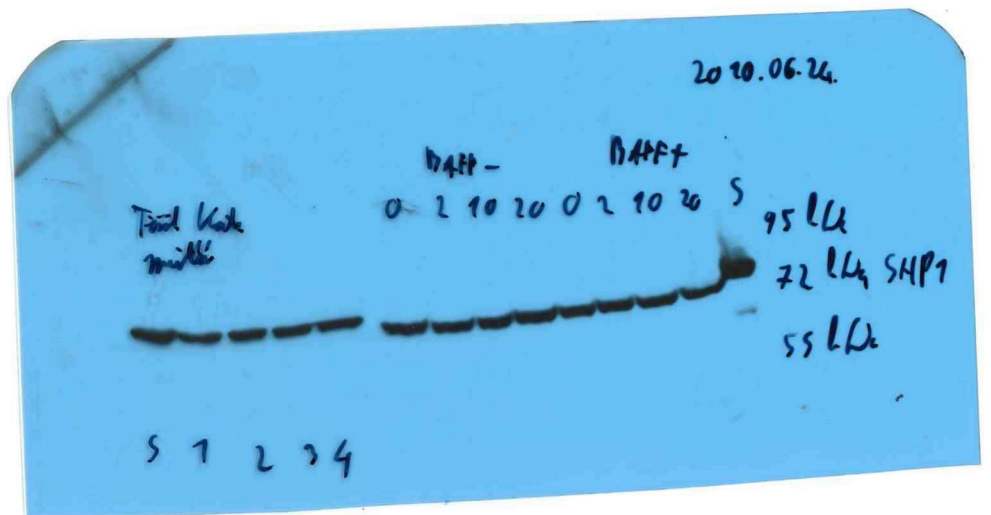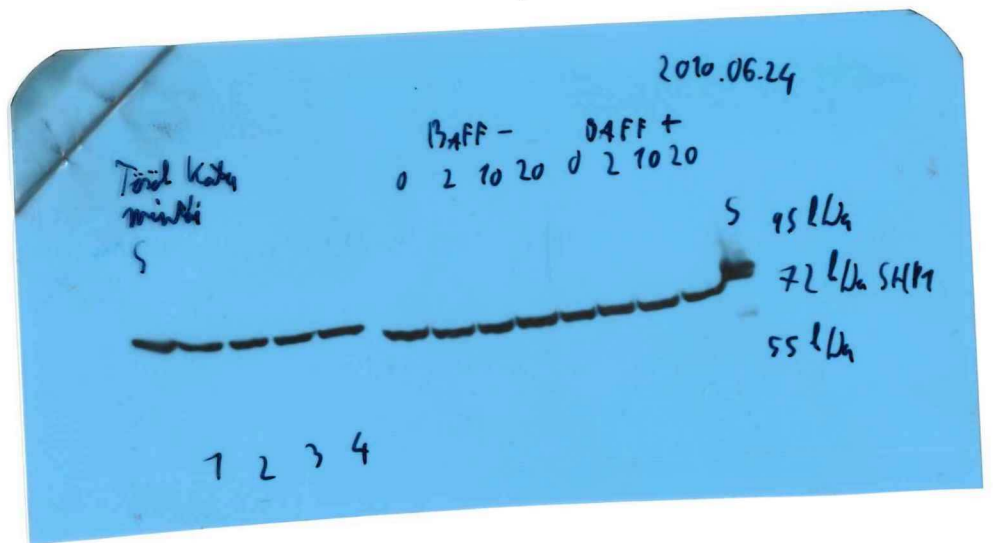

Supplement: S2 File — (PDF) [file pone.0265030.s002.pdf]

2010.06.24

DAFF+

10 12 120 C0 C2 C20 100 102 100 S

SHP1

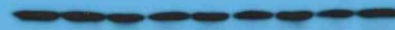

72 kDa

2010.06.24

DAFF+

10 12 120 C0 C2 C20 100 102 100 S

SHP1

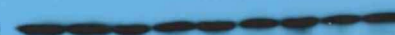

72 kDa

Supplement: S3 File — (PDF) [file pone.0265030.s003.pdf]

2017.08.31.

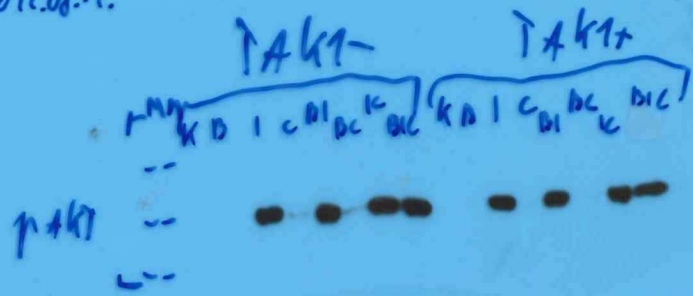

2017.08.31.

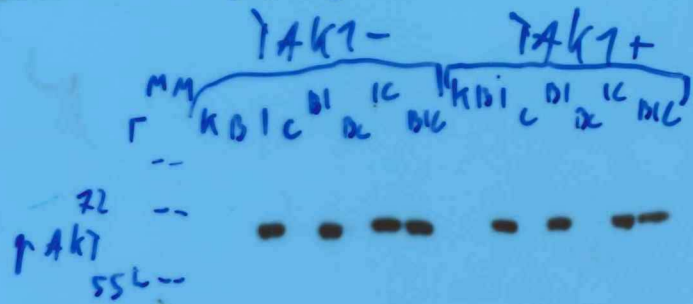

2017.09.02.

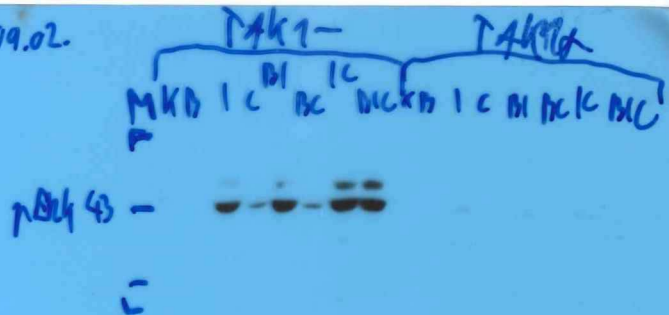

Supplement: S4 File — (PDF) [file pone.0265030.s004.pdf]

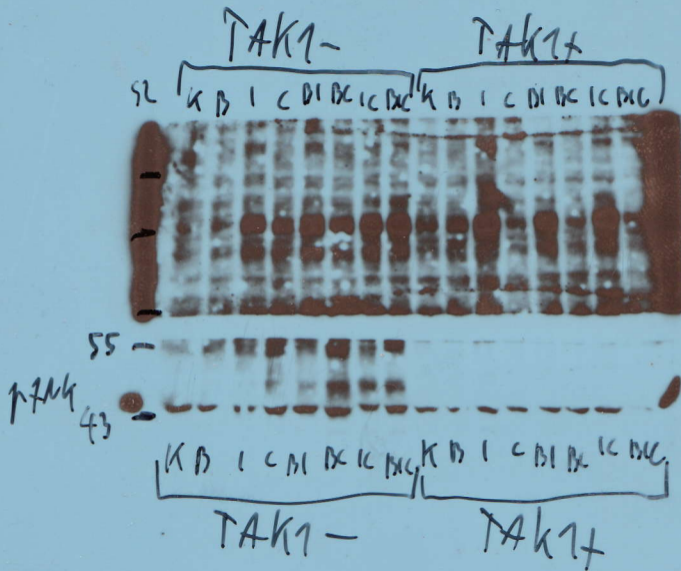

20120827

Supplement: S6 File — (PDF) [file pone.0265030.s006.pdf]

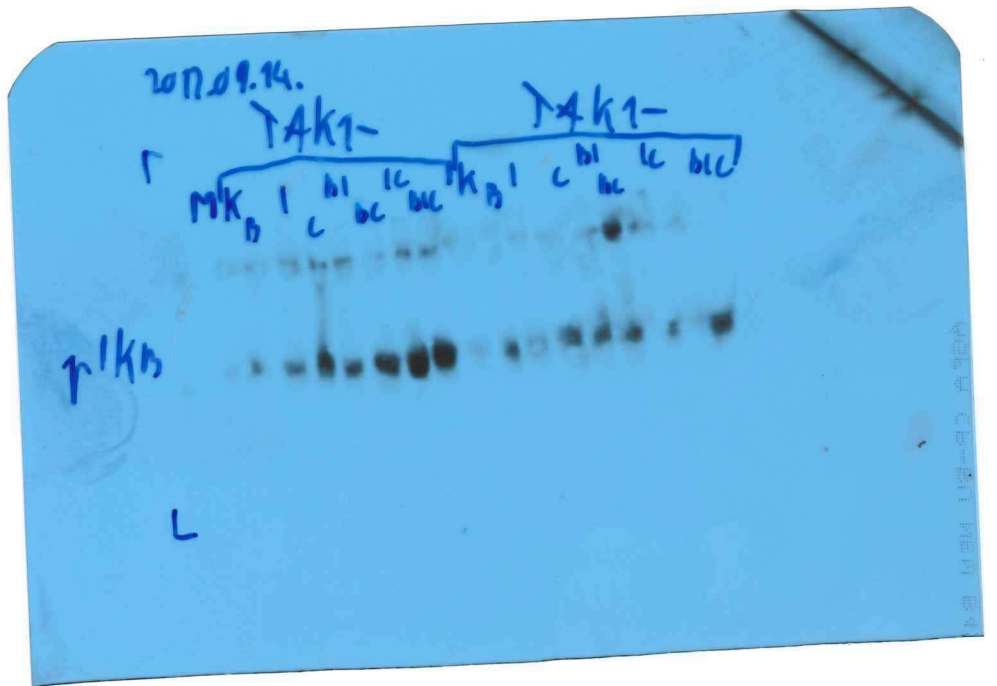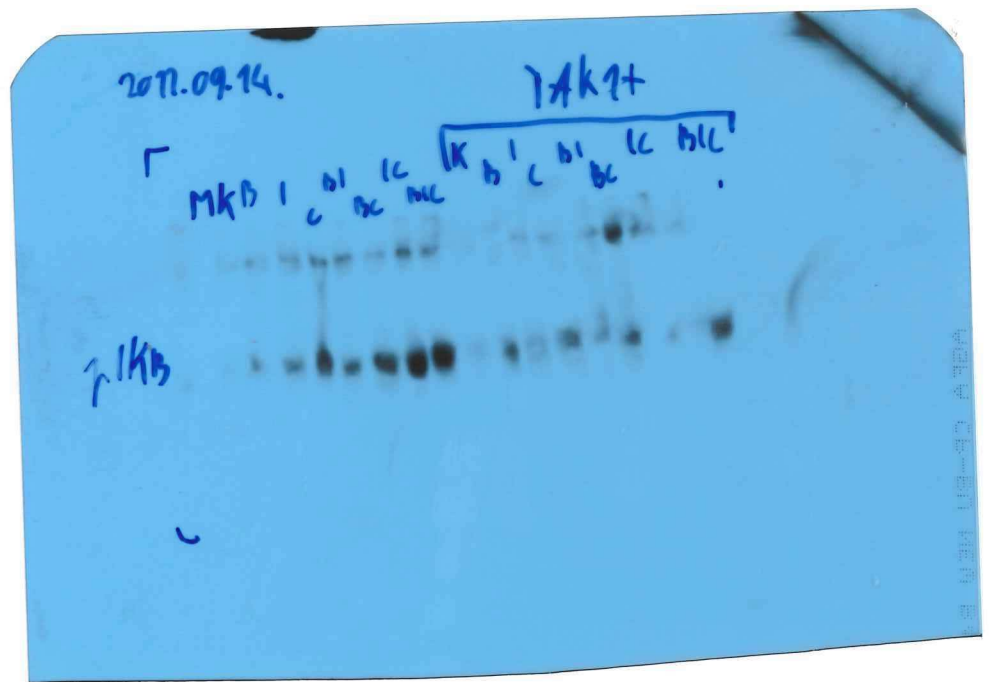

Supplement: S7 File — (PDF) [file pone.0265030.s007.pdf]

2012.08.27

TAK1T  
BK IC DC C T D K  
TAK1-  
BK IC DC C I H K M

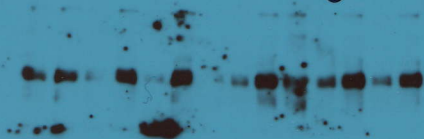

-95 wFoxo  
-72

Supplement: S8 File — (PDF) [file pone.0265030.s008.pdf]

TAK1+

TAK1-

2012.09.01

NC LC BI C IB K NC LC BI C IB K MS

- 95

- 72

SHM

- 55

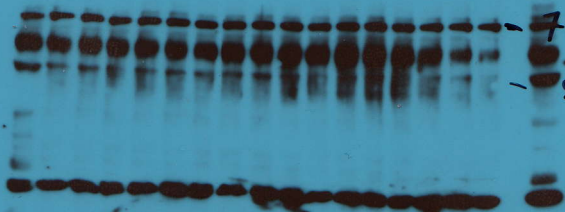

Supplement: S9 File — (PDF) [file pone.0265030.s009.pdf]
