# Supplementary material for: Expression of Concern: TGFβ Activated Kinase 1 (TAK1) at the Crossroad of B Cell Receptor and Toll-Like Receptor 9 Signaling Pathways in Human B Cells
Source: PLoS One. 2022 Mar 3;17(3):e0265030. doi: 10.1371/journal.pone.0265030 (PMC8893696; doi:10.1371/journal.pone.0265030)

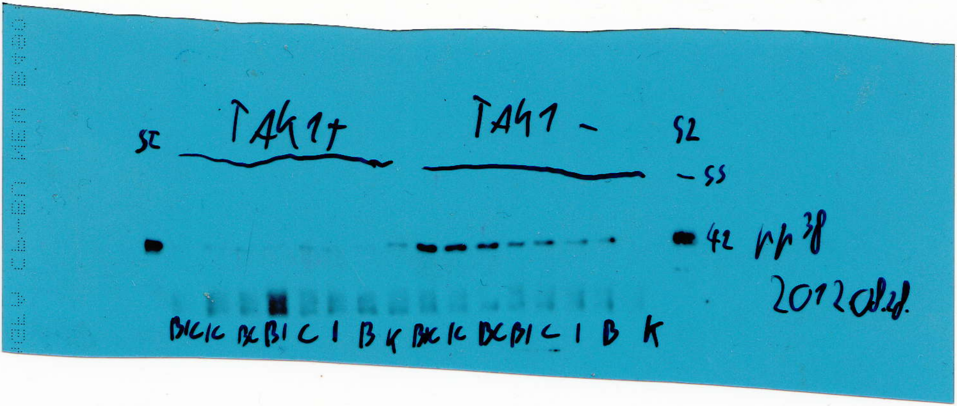
TAK1+
TAK1 -
55
42
pp38

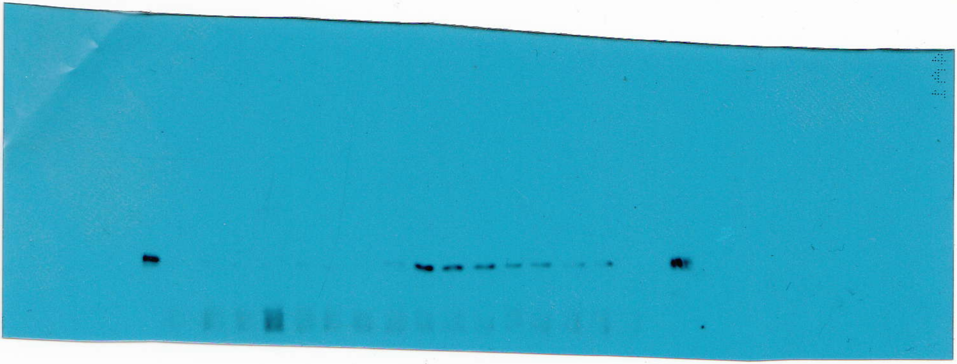

Supplement: S5 File — (PDF) [file pone.0265030.s005.pdf]
